# On the Injurious Effects of Camphor on the Teeth

**Published:** 1847-06

**Authors:** 


*On the Injurious Effects of Camphor on the Teeth.*—Pernicious effects of this agent, when used as a dentifrice, have been recently very positively asserted in our medical journals, and contradicted with not less decision. Experience, however, is in favor of this latter opinion; for, after a long series of experiments by W. Herapath, the distinguished lecturer on chemistry, in the Bristol Medical School, with a view to ascertain whether any, and what, action camphor exerts over human teeth. The experiments were performed on whole teeth, and on sections of teeth, with camphor in water, alcohol and human saliva, at all temperatures, between 98 deg. (blood-heat) and 110 deg. (fever-heat,) and with and without a current of atmospheric air. The experiments were continued for ten days, and the result showed that no injurious action was exercised either on the enamel or the bony structure of human teeth.

A subject deserving of attention, is its influence as an internal agent; for we find that, "at a late meeting of the Societe Medico-Pratique at Paris, many of the members cited facts, tending to prove that camphor is a medicine, the abuse of which is extremely dangerous. M. Homolle related a case of phthisis, in which he prescribed more than twenty grains of camphor, in divided doses, in the twenty-four hours; the effect of which was, that the patient was attacked with frightful dyspnœa, continued nausea, and violent palpitation of the heart, all of which symptoms were with much difficulty subdued. Dr. Gaide mentioned the case of a man who was in the habit of taking camphor in very large doses, as a consequence of which he became affected with aggravated diphtheritis. M. Moreau stated, that he had seen a lady attacked with acute meningitis, which only yielded to the most active treatment, from having taken large doses of camphor to cure an obstinate neuralgic affection. Dr. Labarraque said, that a butcher, for whom he had prescribed six grains of camphor, was attacked with violent vomitings, which nearly proved fatal."—*Abridged from the Dublin Journal.*

